# Reduced health-related quality of life among Japanese college students with visual impairment

**DOI:** 10.1186/s13030-015-0045-1

**Published:** 2015-08-29

**Authors:** Iguchi Masaki

**Affiliations:** Tsukuba University of Technology, Kasuga 4-12-7, Tsukuba, Ibaraki 305-8521 Japan

## Abstract

**Background:**

Although previous studies have shown detrimental effects of visual impairment on health-related quality of life (HRQOL), they were primarily conducted on elderly individuals with visual impairment. The objective of this cross-sectional study was to investigate if HRQOL is impaired in young college students with visual impairment and to explore the relationships between HRQOL and other factors. It was hypothesized that visual impairment is not influential enough to lower the HRQOL of young people due to their better physical fitness and more flexible mentality.

**Methods:**

A total of 21 college students (mean age = 25 years old) with varying degrees of visual impairment completed the short form (SF)-36 health survey and questionnaires on daily physical activities. Subjects were grouped depending on the type of visual impairment: blind (*n* = 11) or severely impaired (*n* = 10). In addition, grip strength and single-leg standing balance were assessed.

**Results:**

No between-group differences were found in the SF-36 scores. However, compared to the general Japanese standards (50.0 ± 10.0), the Vitality scores of the blind group were lower (41.9 ± 7.2, *p* = 0.004) and the Physical Function scores of the severely impaired group were higher (55.3 ± 2.4, *p* = 0.001). In addition, a negative correlation was found between standing balance (variability of foot center of pressure) and the Physical Component Summary score of the SF-36 (r^2^ = 0.35, *p* = 0.005).

**Conclusions:**

These findings suggest that even among young people severe visual impairment leads to reductions in some components of HRQOL.

## Background

Health-related quality of life (HRQOL) is important, because it is determined not by healthcare providers but by those who receive the healthcare, and moreover, it reflects how patients perceive their health status. Diminished visual acuity has been associated with decreased performance of instrumental activities of daily living (ADL), poorer cognitive abilities, increased risk of falls, and ultimately, a poorer HRQOL [1–7]. However, the currently available data were primarily obtained from studies on elderly people with visual impairment. The present study investigated if the detrimental effects of visual impairment on HRQOL are as strong among young people.

Because visual impairment is more common in elderly people [8] and its prevalence is expected to double over the next 30 years because of the aging population [9], most data on HRQOL of visually impaired individuals are obtained from studies on elderly adults [7]. However, aging alone can influence HRQOL, and although the prevalence may be low, there are certainly young adults with visual impairment who study and are working professionals. Therefore, an investigation of HRQOL among young adults with visual impairment would result in a deeper understanding of HRQOL with visual impairment.

Age is the most important variable predicting the decline in the performance of ADL [10], and an association between visual impairment and difficulty in performing ADL has been reported in elderly people [11–15]. Moreover, aging changes how people perceive the status of their health. Older people are more likely to be optimistic in their global self-assessments of health, resulting in better self-assessed health scores [16]. In fact, in very elderly people (>80 years old), the effect of visual impairment on HRQOL has been reported to be milder than in less elderly people [17]. These previous findings suggest that data on young people with visual impairment may be different from those on elderly people.

Some studies have investigated the effects of visual impairment on HRQOL in young people. Visual impairment affects all aspects of a child or young person’s life. Visual impairment has detrimental effects not only on the physical but also on mental domains of HRQOL, resulting in frustration and concerns of the future [18]. On the other hand, the well-established idea known as the “disability paradox” [19, 20], in which people with a severe disability report high QOL and vice versa, appears to apply to children and young adolescents with visual impairment. A previous study reported that many children with severe visual impairment show remarkable psychological adjustment and have ambitious plans and aspirations for the future [21]. However, to the best of the author’s knowledge, there are no studies that investigated the effect of visual impairment on HRQOL among college-age young adults.

Blind individuals have been reported to experience impairment in some physical aspects such as muscle strength and standing balance [22, 23]. Compared to mental functions, these physical functions are more directly impacted by aging. Thus, it may be important to objectively assess some of the physical functions in young people with visual impairment and to explore the relation between physical functions and HRQOL.

The purpose of the present study, therefore, was to determine if HRQOL is impaired in college students with visual impairment. It was hypothesized that, compared to the nation’s standard for HRQOL, visual impairment is not influential enough to lower the HRQOL of young people.

## Methods

### Subjects and general research design

A total of 21 visually impaired college students were recruited via advertisement. None of the subjects had any known disability/impairment other than visual impairment. The subjects were divided into two groups depending on the severity of their visual impairment (based on the Japanese Physical Disability Certificate they possessed): blind (Level 1) or severely impaired but not blind (Level 2). Subjects were considered level 1 when the sum of the visual acuity in both eyes was 0.01 (equivalent to +2.0 logMAR) or less (measured in accordance with the International Visual Acuity Test Chart; the vision of those with refractive errors was measured in relation to corrected vision. The same shall apply hereinafter). Subjects were considered level 2 either when the sum of the visual acuity in both eyes was more than 0.02 (equivalent to +1.7 logMAR) and less than 0.04 (equivalent to +1.4 logMAR) or when the field of vision was less than 10° in both eyes and more than 95 % vision had been lost in both eyes, evaluated according to the visual efficiency scale. The blind and severely impaired groups included 11 and 10 subjects, respectively (with 1 and 2 female, respectively). The causes and onset ages of visual impairment are listed in Table 1. Subjects’ average age, height, and weight were 24.9 ± 6.1 (mean ± standard deviation) years, 165.3 ± 10.1 cm, and 59.0 ± 10.4 kg, respectively, with no between-group differences (independent t-test, *p* > 0.05). All subjects provided written informed consent, and the study protocol was approved by the local Internal Review Board. The study has been conducted in accordance with the guidelines of the 1964 Declaration of Helsinki. The setting for the study was a university laboratory. The data was collected in 2012 and 2013, avoiding the very beginning and very end of school terms in order to minimize any potential influences from school-related events such as exams.

**Table 1:** Demographic data of the subjects

Group	Age (years)	Onset (age in years)	Cause of visual impairment
B1	20	Congenital	Glaucoma
B2	20	Congenital	Retinopathy of Prematurity
B3	26	Congenital	Glaucoma
B4	20	Congenital	Glaucoma
B5	21	Congenital	Glaucoma
B6	21	Congenital	Retinopathy of Prematurity
B7	22	Congenital	Leber Disease
B8	25	Congenital	Retinopathy of Prematurity
B9	24	Congenital	Choroidal Detachment
B10	20	Congenital	Optic Atrophy
B11	23	Congenital	Retinopathy of Prematurity
S1	20	Congenital	Retinal Detachment
S2	28	21	NA
S3	29	Congenital	Retinitis Pigmentosa
S4	37	16	Stevens-Johnson Syndrome
S5	28	Congenital	Retinitis Pigmentosa
S6	39	12	Retinal Detachment
S7	39	Congenital	Retinitis Pigmentosa
S8	22	Congenital	Glaucoma
S9	22	Congenital	Optic Atrophy
S10	20	Congenital	Leber Disease

“B” and “S” indicate blind and severely impaired groups, respectively. There was one subject in the severely impaired group who did not report his/her cause of visual impairment. This is indicated by “NA”

The subjects completed a short form (SF)-36 health survey and a questionnaire about their daily exercise habits. In addition, the grip strength and single-leg standing balance of all subjects were measured.

### HRQOL assessment

The Medical Outcomes Study 36-Item Short-Form Health Survey (SF-36) was used to assess HRQOL. Depending on the subject’s preference, the form was provided in Braille, written regular text, or electronic text. The subjects were asked to complete the form and submit it to the investigator within 1 week.

The SF-36 contains 36 items measuring eight dimensions of health and well-being: “physical function”, “role limitations due to physical problems”, “bodily pain”, “general health perceptions”, “vitality”, “social function”, “role limitations due to emotional problems”, and “mental health” [24]. One item that focused on changes in health was excluded from this study.

### Physical activity questionnaire

All subjects completed a questionnaire on physical activity habits. The amount of daily physical activity was quantified using a method similar to that used in previous studies [25, 26]. Each subject was asked to specify the average number of hours per day spent on each of the following physical activities: strenuous exercise or heavy physical work, walking or standing, and sleeping. This questionnaire was also prepared in the three above-mentioned formats.

### Grip strength measurement

Grip strength was chosen because it represents general muscle strength [27, 28]. The grip strength was measured twice for the dominant hand in a standing position using a Smedley hand dynamometer (T. K. K. 5401, Takei Scientific Instruments Co., Ltd., Niigata, Japan). Two subjects in the blind group were left-handed, and none were left-handed in the severely impaired group. Subjects were asked to grip the dynamometer as hard as they could with their arm hanging by their body and instructed to abduct their shoulder slightly, such that the dynamometer did not touch the lateral aspect of the leg. The investigator gave the subjects verbal encouragement, and a rest of approximately 60 s was taken between trials. The digital numbers were read from the display on the dynamometer, and the highest value of the two trials was used for analysis.

### Standing balance measurement

Standing balance was assessed by center of pressure (CoP) measurement using a Nintendo Wii Balance Board. Its accuracy for CoP measurement has been shown to be acceptable for research [29]. The built-in calibration exports the CoP data in centimeters for anterior–posterior and mediolateral directions at a sampling rate of 100 Hz. The data were stored on a personal computer. The subjects were asked to stand at the center of the board on their dominant leg, defined as the leg used to kick a ball as far as possible (the left leg was dominant in 1 subject in the blind group and none of the subjects in the severely impaired group). The arms were unrestricted but the non-standing leg was not allowed to touch the standing leg. Subjects were asked to stand as stably as possible twice for 15 s each, with a 1-min rest between trials.

### Data analysis

The SF-36 score was calculated according to the instruction manual [30]. The scores for each item were averaged within each of the eight dimensions and within each group and were further converted into norm-based scores using the published data of 2007 Japanese values found in the manual, such that the Japanese mean and standard deviation were 50.0 and 10.0, respectively, in all dimensions. In addition to the general Japanese values, standard values for Japanese males aged between 20 and 29 years old were used for comparison because the majority of the subjects in the present study (86 %) were males, with an average age of 25 years. The physical, mental, and role/social component summary scores (PCS, MCS, and RCS, respectively) were also calculated according to the instructions provided in the manual.

With regard to the physical activity questionnaire, the sum of the time spent on the three activities (strenuous exercise/heavy physical work, walking/standing, and sleeping) was subtracted from 24 (hours) for each subject to obtain the time spent in a sedentary state. Metabolic equivalent (MET) intensities of 4.5, 2.0, 1.5, and 1.0 were assigned for strenuous exercise/heavy physical work, walking/standing, sitting, and sleeping, respectively. Finally, the total MET hours per day was calculated by multiplying the MET and hours for each activity performed and summing them for each subject.

The root mean square (RMS) and peak-to-peak amplitude of the CoP were obtained in each direction (anterior–posterior and mediolateral) after offsetting the data by subtracting the average. The most stable middle 10-s data (visual inspection) of the 15-s data were analyzed.

The norm-based SF-36 scores were analyzed using two-way analyses of variance (ANOVA) to identify any between-group differences in dimensions (2 × 8) and in summary scores (2 × 3), with “group” as the between-subject variable and “dimension” or”summary score” as the within-subject variable. One-sample t-tests were used to compare the values from the present study to the published Japanese values (both the general Japanese values and the standard Japanese values for young males).

Correlation analyses were performed by obtaining Pearson product correlation coefficients between the SF-36 scores and the other variables. To account for multiple comparisons, a significance level of 0.01 was used for all statistical analyses.

In addition, for assessing possible confounding variables and finding models predicting the three component summary scores, a step-wise multiple linear regression analysis with inclusion and exclusion criteria of 0.05 and 0.10, respectively were performed using all the above variables, including age, sex, impairment level, and the onset of impairment. When appropriate, an analysis of covariance (ANCOVA) was also used. The results are presented as mean ± standard deviation or as mean ± standard error (as an error bar) in the text and figures, respectively.

## Results

### SF-36 scores between groups and comparison with published Japanese standards

**Fig. 1:**
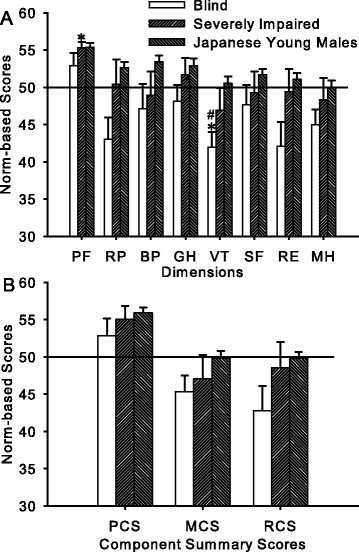
SF-36 scores. **a** The SF-36 norm-based scores in each dimension for the blind group, severely impaired group, and the published Japanese standard for young males. The solid horizontal line at a norm-based score of 50 indicates the general Japanese standard (with a standard deviation of 10). No between-group (blind vs. severely impaired subjects) differences were found. The Physical Function scores of the severely impaired subjects and the Vitality scores of the blind subjects were higher and lower, respectively, than the general Japanese standards [50; indicated by asterisks (*)]. The Vitality scores of the blind subjects were also lower than the Japanese standard for young males [indicated by the number sign. (#)]. **b** Norm-based SF-36 scores in each component summary score. The horizontal line and legends are the same as in (A). No differences were found in any of the comparisons. PF: Physical Function, RP: Role Physical; BP: Bodily Pain; GH: General Health; VT: Vitality; SF: Social Function; RE: Role Emotional; MH: Mental Health; PF: Physical Function; PCS, MCS, and RCS: Physical, Mental, and Role/Social Component Summary Scores, respectively

No between-group differences were found in norm-based SF-36 scores for any of the eight dimensions [Fig. 1a; *F*(1, 19) = 2.60, *p* = 0.12] or for any of the three summary scores [Fig. 1b; *F*(1, 19) = 2.881, *p* = 0.11].

None of the summary scores differed from the general Japanese standards (50.0 ± 10.0) in either group, except for two of the eight dimensions. The Vitality score of the blind group (41.9 ± 7.2) was lower [*t*(10) = −3.69, *p* = 0.004] and the Physical Function score of the severely impaired group (55.3 ± 2.4) was higher [*t*(9) = 6.90, *p* < 0.001] than the general Japanese standards.

Because the majority of the subjects in the present study were young males, the scores were also compared to the Japanese standards for young males. Because the standard Japanese Physical Function score for young males is higher (55.4 ± 5.6) than the general score (50.0 ± 10.0), the Physical Function score of the severely impaired group in the present study was no longer different from the standard score for young males. In contrast, the Vitality score for the blind group remained lower than the Japanese standard for young males [50.5 ± 10.2; *t*(10) = −3.92, *p* = 0.003].

### Between-group comparisons in grip strength, physical activity level, and standing balance

There were no between-group differences in grip strength [*t*(19) = −1.42, *p* = 0.17], physical activity level [*t*(19) = 1.40, *p* = 0.18], or standing balance {p values ranging from 0.34 [*t*(19) = 0.99 for RMS amplitude of the CoP in anterior–posterior direction] to 0.83 [*t*(19) = 0.21 for the RMS amplitude of the CoP in mediolateral direction]}.

### Correlations between SF-36 scores and other variables

**Fig. 2:**
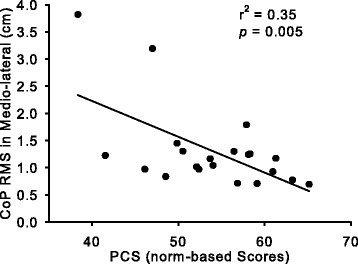
Correlation between SF-36 score and standing balance. The relation between the Physical Component Summary Score (PCS) from the SF-36 and the root mean square (RMS) of the foot center of pressure (CoP) trajectory in the mediolateral direction, using data of all subjects (*n* = 21). Those who had a higher PCS were more stable during single-leg stance

Among the potential correlations analyzed, two significant correlations were found. The average of the two measurements for the CoP in the mediolateral direction, expressed as peak-to-peak (r^2^ = 0.31, *p* = 0.009; data not shown) and RMS (r^2^ = 0.35, *p* = 0.005; Fig. 2) was negatively correlated with the PCS.

### Predicting models using multiple regression and analysis of covariance

In order to assess any confounding factors, multiple linear regression analysis was used. However, the result was the same as that from the simple correlation above. The model predicting PCS had the CoP in the mediolateral direction expressed as RMS. For MCS, the physical activity level remained in the model, but the p value was only 0.040, which was above the significance level set in the present study. These data suggest that no covariates can be used for ANCOVA to find between-group differences in the SF-36 scores.

## Discussion

The current study aimed to determine if HRQOL, as measured by the SF-36, is impaired even in young adults with visual impairment. The novel findings of the present study are as follows: 1. being blind or severely visually impaired did not differentially influence the subjects’ HRQOL; 2. the Vitality score of the blind subjects in this study was lower than the published Japanese standard for a similar age; and 3. although the severity of visual impairment did not influence the physical functions measured in this study (e.g., grip strength), subjects who scored higher in the Physical Component of the HRQOL had better single-leg stance balance than those who scored lower in the Physical Component of the HRQOL. The hypothesis that young age diminishes the detrimental effects of visual impairment on HRQOL is partially supported.

### Influence of aging and visual impairment on HRQOL

It has been clearly demonstrated that visual impairment has detrimental effects on the HRQOL of elderly people and that limitations in performing ADL are associated with a lowered HRQOL [10]. These previous findings suggest that young adults who have fewer limitations in performing ADL should have a better HRQOL than their elderly counterparts. The effect of aging has been reported to have a clear detrimental influence on the physical rather than mental domains of HRQOL [30, 31]. The lack of significant differences in the physical domains between young adults with visual impairment and the standard Japanese scores for young males found in the present study (Fig. 1a) indicate that visual impairment, even blindness, does not inhibit the beneficial effect of being young on the physical domains.

Some questions within the Physical Function dimension ask about the ability of responders to perform vigorous activities such as running and playing strenuous sports. It is obvious that visual impairment would limit participation to only some sporting activities, especially those not using a ball. Most of the 10 items in the Physical Function dimension, however, ask about basic ADL such as bathing and climbing stairs.

In contrast to the effect of aging on HRQOL, it has been reported that visual impairment has a greater impact on the mental rather than physical domains in elderly people [32]. Although the MCS scores of subjects in the present study were not different from the Japanese standards for young males (Fig. 1b), the Vitality score of the blind subjects was lower not only than the general Japanese standard but also than the Japanese standard for a similar young age (Fig. 1a). The items in the Vitality dimension ask how energetic or fatigued responders feel. Although aging does not have a great impact on the scores in the mental domains, including the Vitality score [30], it has been reported that the scores are sensitive to the impact of disease and the treatment of hypertension [33], AIDS [34, 35], and visual impairment [32]. However, the sensitive Vitality score was similar between the blind and severely impaired groups in the present study, a finding that is contrary to that of a previous study [32] in which correctable and non-correctable visual impairments were compared. The non-significant between-group difference in the Vitality score in the present study is probably due to the small sample size (see Limitations below).

### Relation between HRQOL and standing balance

The results of the present study indicate that although the physical functions, such as grip strength, were similar between the blind and severely impaired groups, a correlation was found between balance and the Physical Component Summary score. It has been reported that blind individuals have diminished standing balance compared to that of individuals with unimpaired vision [22, 23]. This is because vision is important in stabilizing the body while standing, and it appears that long-term visual information loss such as that seen in congenitally blind adults cannot be substituted by other sensory inputs necessary to stabilize the body, such as the vestibular system [23]. The relation between balance and the physical component of HRQOL also has been reported in stroke patients [36]. These previously reported data together with those from the present study suggest that standing balance is critical and that balance training could serve as a method to improve the physical components of HRQOL among visually impaired people.

### Limitations

There are many limitations to this study. The two major limitations are the small sample size, which reduced the statistical power, and that all the subjects were college students, which could have resulted in sampling bias. Therefore, the results are not necessarily applicable to all young adults with visual impairment. However, studying at college is common in developed countries for people in their twenties. Recruiting all the subjects from college would generate consistent data, which may explain why the multiple regression analysis and ANCOVA were not applicable here. The causes and onset ages of visual impairment varied among the subjects; however, it is unlikely that different causes greatly affected the results because it has been reported that the severity of, but not the cause of, visual impairment impacts HRQOL [37]. In the present study, no control data were collected from young college students with no visual impairment. Instead, published data was used. Ideally, the groups should be as similar as possible, but the advantage of using the published data is its large sample size (*n* = 117 for the data used here). No information about past history of physical/mental diseases were obtained from the subjects, and therefore, the data obtained here could have been affected by the past history of diseases. Finally, generic health outcome measures such as the SF-36 have been reported to be less sensitive to ocular conditions compared to vision-related health outcome measures such as the Visual Functioning-14 [7]. However, some differences were detected using the less sensitive measure.

## Conclusions

Because aging and visual impairment are both strong negative factors for HRQOL, the present study evaluated if the HRQOL of young college students with visual impairment is lower than that of persons of a similar age in the general population, as is observed in elderly individuals with visual impairment [32]. The data suggest that the Vitality score only of blind subjects was lower than the standard. In addition, how well a young subject with visual impairment could stand on a single leg predicted how high the subject would score on the Physical component of HRQOL. These data suggest that for visually impaired people being young and having good balance are important factors for better HRQOL.
