# Erratum to: Reduced health-related quality of life among Japanese college students with visual impairment

**DOI:** 10.1186/s13030-016-0061-9

**Published:** 2016-04-05

**Authors:** Masaki Iguchi

**Affiliations:** Tsukuba University of Technology, Kasuga 4-12-7, Tsukuba, Ibaraki 305-8521 Japan

After publication of this article [1], the author realised their family name and given name had been incorrectly reverted in the author list. Their name was incorrectly presented as Iguchi Masaki when it should be Masaki Iguchi. The correct presentation of the author’s name is included in the author list of this erratum.
